# Accuracy of models for the 2001 foot-and-mouth epidemic

**DOI:** 10.1098/rspb.2008.0006

**Published:** 2008-03-25

**Authors:** Michael J Tildesley, Rob Deardon, Nicholas J Savill, Paul R Bessell, Stephen P Brooks, Mark E.J Woolhouse, Bryan T Grenfell, Matt J Keeling

**Affiliations:** 1 Department of Biological Sciences and Mathematics Institute, University of WarwickGibbet Hill Road, Coventry CV4 7AL, UK; 2 Department of Mathematics and Statistics, MacNaughton Building, University of GuelphGuelph, Ontario, Canada N1G 2W1; 3 Veterinary Epidemiology Group, Centre for Infectious Diseases, University of EdinburghEaster Bush Veterinary Centre, Roslin, Midlothian EH25 9RG, UK; 4 Statistical Laboratory, Centre for Mathematical Sciences, University of CambridgeWilberforce Road, Cambridge CB3 0WB, UK; 5 Center for Infectious Disease Dynamics, Biology Department, Pennsylvania State University208, Mueller Laboratory, University Park, PA 16802, USA

**Keywords:** foot and mouth, model–data comparison, stochastic, spatial

## Abstract

Since 2001 models of the spread of foot-and-mouth disease, supported by the data from the UK epidemic, have been expounded as some of the best examples of problem-driven epidemic models. These claims are generally based on a comparison between model results and epidemic data at fairly coarse spatio-temporal resolution. Here, we focus on a comparison between model and data at the individual farm level, assessing the potential of the model to predict the infectious status of farms in both the short and long terms. Although the accuracy with which the model predicts farms reporting infection is between 5 and 15%, these low levels are attributable to the expected level of variation between epidemics, and are comparable to the agreement between two independent model simulations. By contrast, while the accuracy of predicting culls is higher (20–30%), this is lower than expected from the comparison between model epidemics. These results generally support the contention that the type of the model used in 2001 was a reliable representation of the epidemic process, but highlight the difficulties of predicting the complex human response, in terms of control strategies to the perceived epidemic risk.

## 1. Introduction

Mathematical modelling of infectious diseases has recently progressed from a descriptive to predictive science that can be used as a potential public health or veterinary tool. The success of such models can be traced from the early work on rubella vaccination (Anderson & May 1983) through the 2001 UK foot-and-mouth epidemic (Ferguson *et al*. 2001*a*; Keeling *et al*. 2001; Morris *et al*. 2001) where models were used in real time. More recently, models have been used to assess the epidemic potential and control mechanisms against smallpox (Ferguson *et al*. 2003; Hall *et al*. 2007) and pandemic influenza outbreaks (Longini *et al*. 2004; Ferguson *et al*. 2005). One considerable challenge to any modelling study is parametrization, in particular assessing the many unknown and unmeasurable parameters that allow the model to capture the observed outbreak. The other, but related, issue is to determine reliable and meaningful statistics to compare the detailed model output with the epidemic data.

Here, we perform a detailed analysis on the models of Keeling *et al*. (2001, 2003) in comparison with the 2001 foot-and-mouth epidemic data. In particular, although these models were parametrized to match aggregate regional data (as explained below), we assess the ability of such models to predict the status of individual farms. The 2001 foot-and-mouth disease (FMD) outbreak is exceptional in terms of the detailed spatio-temporal information available for the epidemic cases and culls and the information on the distribution of initial susceptible farms. This allows for detailed spatio-temporal stochastic models that operate at the farm level to be developed and parametrized. We start by briefly reviewing the 2001 epidemic and the available information, followed by the model of Keeling *et al*. We next consider a suitable measure of accuracy, which captures the agreement between model predictions and data, before finally commenting on the model's suitability and potential for improvement.

## 2. 2001 FMD epidemic and data

Numerous accounts of the 2001 FMD epidemic in the UK have been published (e.g. Anderson 2002; Kitching *et al*. 2005). Here, we outline the salient factors that impact on the model formulation and the comparison between the observed epidemic and the model output. In essence, before the 2001 epidemic, there were 188 496 farms identified as containing livestock, although only 142 496 farms were part of the June 2000 census. For all 188 496 livestock farms that act as the susceptible denominator for the epidemic, we know the County–Parish–Holding number (CPH), the *X*- and *Y*-coordinates of the farmhouse, the area of the farm and the number of cattle, sheep, pigs, goats and deer, although these livestock quantities are subject to variation during the year as new animals are born and older animals moved on or off the holding.

Data on the holdings that were culled as part of the FMD control measures were stored in the Department for Environment Food and Rural Affairs Disease Control System database. Culled holdings were placed into one of the following three categories (Anderson 2002).

- *Infected premises* (*IP*). A holding that was diagnosed positive for foot-and-mouth disease virus (FMDV) on either clinical diagnosis or laboratory analysis of the tissue and/or serum of one or more animals. A total of 2026 IPs were identified on the UK mainland. In many cases, a holding was declared an IP solely on clinical grounds, although tissue or fluid samples were sent to the FMD World Reference Laboratory in Pirbright for confirmation; as a result, 1720 samples from IPs were tested for FMDV and 1320 (76.7%) confirmed as positive for antibody or antigen (Ferris *et al*. 2006).
- *Dangerous contacts* (*DCs*). This category includes all holdings in which no evidence was found indicating the presence of FMDV, but it was believed that the holding was at elevated risk of becoming infected. Reasons for being declared a DC include holdings that had been linked to an IP via epidemiological tracing and, from late March, holdings that were contiguous to an IP—these were often referred to as contiguous premises (CPs). Also, in late March in Cumbria and Dumfries and Galloway, sheep flocks that ‘may be harbouring the disease’ were culled under the 3 km cull (Thrusfield *et al*. 2005). The entire stock on a DC was not always culled if it was judged that not all stock had been exposed to infection (Honhold *et al*. 2004). In some cases, serum samples were taken from animals on DCs, and if they were found to be positive, the DC would be reclassified as an IP.
- *Slaughter on suspicion* (*SOS*). Introduced on 24 March 2001 to include holdings on which clinical symptoms were indecisive (Anderson 2002). The holding would be culled and reclassified as an IP if the holding tested positive for FMDV upon serological testing.

During the 2001 epidemic, there were a total of 1423 DCs, 3619 CPs, 2980 3 km culls and 280 local culls. A total of approximately 3.5 million sheep, 592 000 cattle and 143 000 pigs were slaughtered and recorded in the DCS; in addition, 1.8 million sheep, 166 000 cattle and 306 000 pigs were culled for welfare purposes (Anderson 2002). However, the animals that were part of the welfare cull were not recorded as part of the culled holdings list that we use throughout this paper—previous results have indicated that welfare culls had a minimal impact on the progress of the epidemic (Keeling *et al*. 2001).

### (a) A stochastic spatial FMD model

The model used throughout this paper is an adaptation of a model developed by Keeling *et al*. (2001), and is used to study the effects of various control options (Keeling *et al*. 2003; Tildesley *et al*. 2006). The epidemiological part of the model takes a relatively simple form; the rate at which an infectious farm *i* infects a susceptible farm *j* is given by

$${\text{rate}}_{ij}=\left({\left[N_{\text{sheep},j}\right]}^{p_{\text{s}}}S_{\text{sheep}}+{\left[N_{\text{cow},j}\right]}^{p_{\text{c}}}S_{\text{cow}}\right)\times \left({\left[N_{\text{sheep},i}\right]}^{q_{\text{s}}}T_{\text{sheep}}+{\left[N_{\text{cow},i}\right]}^{q_{\text{c}}}T_{\text{cow}}\right)\times K(d_{ij}),$$

where *N*_*s*,*i*_ is the number of livestock species *s* recorded as being on farm *i*; *S*_s_ and *T*_s_ measure the species-specific susceptibility and transmissibility; *d*_*ij*_ is the distance between farms *i* and *j*; and *K* is the transmission kernel, estimated from contact tracing, which captures how the rate of infection decreases with distance (Keeling *et al*. 2001). The model parameters are determined for five distinct regions, Cumbria, Devon, the rest of England (England excluding Cumbria and Devon), Wales and Scotland, which enable us to account for regional variation in culling levels and farming practices. For each region, this model has seven parameters that need to be estimated (*S*_cow_, *T*_sheep_, *T*_cow_, *p*_s_, *p*_c_, *q*_s_ and *q*_c_, with *S*_sheep_=1). As an improvement to the previous versions of this model (Keeling *et al*. 2001, 2003), the number of livestock is now raised to powers (*p* and *q*) to account for the nonlinear increase in susceptibility and transmissibility of a farm with increasing numbers of animals. The seven unknown parameters are estimated by fitting the model to the aggregate regional time-series data, as explained below. This extra detail is found to improve the overall fit and accuracy of the model, but does not qualitatively change any of the conclusions of this paper.

Two types of culling strategy are modelled: DCs and CPs. During the 2001 epidemic, DCs were identified for each IP on a case-by-case basis, and were based on veterinarian judgement of risk factors and known activities, such as the movement of vehicles. In our model, DCs are determined stochastically, such that the probability that farm *i* is a DC associated with IP *j* is given by

$$\{\begin{array}{c} 1-f\;\text{exp}\;(-F\;{\text{rate}}_{ij})\;\text{if}\;i\;\text{has}\;\text{been}\;\text{infected}\;\text{by}\;j\\ 1-\text{exp}\;(-F\;{\text{rate}}_{ij})\;\text{otherwise} \end{array}.$$

The parameter *f* controls the accuracy of DC culling—the ability to detect the routes of transmission—while *F* governs the overall level of DC culling per reported case; *F* is allowed to vary through time to reflect the changing levels of DC culling that occurred during the epidemic (Tildesley *et al*. 2006), while *f* is another free parameter that needs to be estimated. We use the same spatial kernel in determining infection and the identification of DCs, although in principle, it may be possible to estimate different kernels reflecting any biases in DC ascertainment.

CPs are identified in the model by tessellating around each farm location, taking into account the known area of each farm, to obtain a surrogate set of adjacent farms. During 2001, CPs were determined by a more comprehensive knowledge of the farm geography and are defined as farms that share a common boundary—in practice this was determined on a case-by-case basis using local maps and knowledge. Many premises in the UK comprise multiple parcels or fragments of land. It has been argued that fragmentation of farms was a risk factor in the 2001 epidemic (Ferguson *et al*. 2001*b*), although highly fragmented farms were generally contiguous to a greater number of farms and therefore more likely to be culled as a CP. The effect of this can be seen from the cellular automata model of Kao (2003). Some farm fragments have their own unique CPH number in the census database and the tesselation method will explicitly calculate CPs for these fragments. However, for all other farms, we make the simplifying assumption that each farm is made up of one parcel of land. Clearly, the CPs predicted by the tesselation will not necessarily correspond to the true set of CPs, particularly when considering farms comprising multiple parcels. However, this method of estimating CPs will capture many of the elements of local proximity (Keeling *et al*. 2001). The extent of CP culling is captured by a single time-varying parameter that reflects the ratio of CP culls to IPs that occur at any point in the epidemic.

During the 2001 epidemic, many other types of culling were performed including 3 km culls, SOS and local culls. Such culls are difficult to model explicitly, as their timing and implementation is often contingent on non-epidemic factors such as perceived risk. Given the general localized nature of these other forms of cull, they have been incorporated within the model into the DC culling strategy, modifying the values of *F* and *f*. In particular, the 3 km cull in Scotland and Cumbria is not explicitly modelled. However, given that distance is the predominant risk factor for DCs, a temporary increase in the levels of DC culling during the times of the 3 km cull provides a reasonable approximation of this spatially localized control. We stress, however, that the level of all culls (including DC and CP culls) is strongly influenced by the human response to the epidemic, and is therefore likely to be a function of the overall epidemic history; for a different stochastic realization, the pattern of culls could deviate significantly from the timing in 2001. As the welfare culls are not recorded in the list of culled holdings described above, we do not attempt to model welfare culling in this paper.

As mentioned above, the data and model parameters are split into five distinct regions. For each region, parameters are found that minimize the average difference between simulated epidemics from the model and the observed 2001 data for the cumulative number of farms reported and culled as well as the cumulative number of cattle and sheep on such farms. This is achieved through repeated simulation. More precisely, in each region we seek to minimize

$${\text{error}}^{2}=\sum_{X\in \xi }\sum_{t=23\;\text{February}}^{1\;\text{October}}{\left[\frac{C_{t}(X_{2001})-C_{t}(X_{\text{model}})}{C_{1\;\text{October}}(X_{2001})}\right]}^{2},$$

where *ξ*={reported farms, culled farms, sheep on reported farms, sheep on culled farms, cattle on reported farms, cattle on culled farms} and *C*_*t*_(*X*) gives the cumulative amount of quantity *X* up to time *t*. Intuitively, we seek to minimize the relative differences between the model and the data in terms of both farms and animals; cumulative rather than daily values are used as these are less affected by small discrepancies in the precise timing of events. It is important to note that this fitting procedure only matches to aggregate statistics at the regional level, and information on the precise farms involved is not used; therefore, the comparison between the model and the data at the individual farm level described below acts as an independent test of the model accuracy.

The estimated model parameters for all regions are given in table 1. The differences in the parameters between the regions are a partial reflection of differences in farming practices in different areas of the country; however, some of the differences are attributable to the way that the nonlinear behaviour with animal numbers on a farm is approximated and the differing distributions of animal numbers in each region.

## 3. Measures of agreement

When attempting to fit models to data, the most statistically rigorous methodology is to calculate the likelihood of the model producing the observed epidemic, i.e. the probability of the model generating exactly the same farms infected and culled on exactly the same days as occurred during the real epidemic. However, while such probabilities are at the heart of many mechanisms of statistical parameter estimation, there are two difficulties with such a measure of accuracy. First, the minuscule probabilities of generating an identical epidemic are difficult to judge intuitively and therefore difficult to communicate to a non-mathematical audience. Second, it is not clear that the likelihood probability captures what is intuitively felt to be the ‘accuracy’ of a model; from a practical perspective, one of the measures of interest to policy makers is robust prediction of general patterns of infection several weeks in the future. For this reason, we consider the accuracy of our model using a variety of statistical measures, predominantly focusing on the ability to correctly identify cases and culls in medium- to long-term predictions.

Figure 1 shows the log likelihood of correctly predicting the status of all the UK farms in short (one week) simulations, for varying start dates. The start date varies in weekly increments and simulations run forward for a period of one week after which time the model and 2001 data are compared. We note that the log likelihood scales linearly with the number of reported and culled farms (see the inset), suggesting a consistent probability of correctly identifying the status of each farm throughout the epidemic. In general, however, these log likelihood values are strongly influenced by the few cases or culls in each week, which occur with extremely low probability. These farms are often small or at some distance from the prevailing epidemic. While such likelihood methods are undoubtedly very powerful tools for giving a comprehensive measure of the global accuracy of a model, we now examine a range of more simplistic measures. In particular, we focus on the average proportion of cases and culls which can be correctly identified by simulations of various lengths for a range of initial starting dates.

We start by defining a matrix of nine variables, which captures the status of farms in both the observed epidemic and the model simulations. *N*_*XY*_(*X,Y*∈{R, C, S}, for reported, culled and susceptible) gives the number of farms that are in state *X* for the observed 2001 epidemic and state *Y* in the model simulation. Thus, *N*_RC_(=733) counts all farms that reported infection during 2001, but were predicted by the simulation to be culled (CP, DC or extended cull) as part of the control measures. We emphasize that we consider reported cases and not infection as only the former can be accurately ascertained from the 2001 epidemiological data: some infected farms will be culled before they report and some reported cases may be misdiagnosed. The matrix of *N* values can then be averaged over multiple realizations of the simulated epidemic (table 2).

The simplest measure of model accuracy is to calculate the proportion of farms that are predicted by the model to be in the same final state as observed in 2001

(3.1) $$\text{accuracy}=\frac{N_{\text{SS}}+N_{\text{RR}}+N_{\text{CC}}}{\text{total}\;\text{number}\;\text{of}\;\text{farms}}.$$

From multiple model simulations (begun on 23 February and iterated until the epidemic dies out), we calculate the accuracy to be 92.46% (95% of simulations lie within 91.65–93.16%). This value indicates that, countrywide and for the entire epidemic, models initiated with the conditions on 23 February can correctly identify the final status of individual farms with a high level of precision.

While this formulation provides a measure of total accuracy, this simple single-valued definition fails to provide sufficient information about the causes of any inaccuracy and is heavily weighted by the success of predicting susceptible farms in the disease-free regions. We therefore partition the accuracy in terms of times and classes of farm considered. ${\text{accuracy}}_{\text{type}}(t_{0},t_{\text{e}})$ is calculated from simulations using the known conditions at time *t*_0_ and iterated until time *t*_e_; comparisons between the model and data are then made at time *t*_e_ and are restricted to those farms in class type in the data that were unaffected (neither culled nor reported infection) at time *t*_0_. Therefore, accuracy_Cumbria_ (23 February, End) calculates the proportion of farms in Cumbria, which have the same status in the model and data at the end of the epidemic, while accuracy_reported_(23 February, 23 March) calculates the proportion of reported farms during the first month of the epidemic, which are correctly identified by the model.

To provide some guidance as to the expected level of between-epidemic variability that places a natural upper bound on the accuracy, we calculate a similar measure comparing the results of two independent (but identically parametrized) model simulations. This measure, which we call repeatability, essentially captures how well the model can predict itself—a high level of repeatability shows that there is little between-epidemic variability at the individual farm level, whereas low level of repeatability shows that between-epidemic variability within the model is high. The repeatability should always be higher than the accuracy, and similar levels of repeatability and accuracy indicate good parametrization of the model at the individual level, given the constraints of the modelling framework.

Here, it is worth stressing the three important points about these measures of model fit. The first is that accuracy measures that focus solely on reported cases or culls (e.g. accuracy_reported_) are only informative if the number of cases and culls in the model closely matches the data. For example, very high levels of reported case accuracy could be obtained if the model simply overestimated the number of cases. We note however that our model closely matches the temporal pattern of observed cases and culls (figure 2*b*). Second, comparable levels of accuracy and repeatability can be achieved when the model captures little of the observed spatial structure—when the model matches the temporal dynamics but not the spatial. However, we again note that our model has been shown to be a good match for the general spatial pattern of cases (Keeling *et al*. 2001). Finally, accuracy_reported_ can be thought of as the sensitivity of the epidemiological prediction; however, as this quantity varies both spatially and temporally and depends on the prediction of culls, we retain the term accuracy. Similarly, we can calculate the specificity of the epidemiological prediction (assuming the model on average predicts the observed number of reported cases)

$${\text{specificity}}_{\text{reported}}=\frac{\text{number}\;\text{of farms}-\text{number}\;\text{of reported}\times {\text{accuracy}}_{\text{reported}}}{\text{number}\;\text{of farms}-\text{number of}\;\text{reported}}.$$

Hence, our measure of accuracy naturally encompasses some of the standard measures of agreement between models and data.

While our accuracy measure provides an intuitive concept of the precision with which the status of individual farms can be detected, it is informative to relate these results to odds ratios, which is an alternative method for assessing the goodness of fit. As such, the odds ratio informs us whether the model results provide a useful predictive diagnostic for the status of a farm. The odds ratio *O*_r_ can be defined in terms of the number of farms of a particular status in the model and data

(3.2) $$O_{\text{r}}=\frac{N(D_{+\text{ve}},M_{+\text{ve}})\times N(D_{-\text{ve}},M_{-\text{ve}})}{N(D_{-\text{ve}},M_{+\text{ve}})\times N(D_{+\text{ve}},M_{-\text{ve}})},$$

where *D* and *M* refer to the statuses of the farms in the model and the data, respectively. Therefore, if we are considering the odds ratio for reported farms, *N*(*D*_+ve_, *M*_+ve_)=*N*_RR_ corresponds to the numbers of farms that are reported in both the data and the model, whereas *N*(*D*_+ve_, *M*_−ve_)=*N*_RS_+*N*_RC_ corresponds to the number of farms that are reported in the data but not reported in the model. Using the matrix of values as illustrated in table 2 leads to the odds ratio for reported farms taking the form

(3.3) $$O_{\text{r}}(\text{reported})=\frac{N_{\text{RR}}\times (N_{\text{SS}}+N_{\text{CC}}+N_{\text{SC}}+N_{\text{CS}})}{\left(N_{\text{SR}}+N_{\text{CR}})\times (N_{\text{RS}}+N_{\text{RC}}\right)}.$$

The higher the odds ratio, the better the model is at predicting the status of the farms in the 2001 epidemic.

We can also relate the odds ratio to the original accuracy measure. Again, considering only the success of capturing reported farms and making the simplifying assumption that the model accurately captures the number of reported farms, we have

(3.4) $$O_{\text{r}}(\text{reported})={\text{accuracy}}_{\text{reported}}\left(\frac{\text{no.}\;\text{of farms}-\text{no.}\;\text{of reported}\times (2-{\text{accuracy}}_{\text{reported}})}{1-{\text{accuracy}}_{\text{reported}}}\right).$$

Hence, for the levels of accuracy observed from our model, it is reasonable to assume that the odds ratio scales approximately linearly with the level of accuracy.

Finally, as with the accuracy measure, we also wish to compute an odds ratio for the ability of the model to predict the results of a model simulation. This model–model comparison again provides an upper bound for the model–data comparison, with a close agreement between the two suggesting that the model is capturing the data as can be expected.

## 4. Comparison of model and data

Figure 2 shows a range of comparisons between the 2001 epidemic data and the model simulations. Figure 2*a* shows the daily number of reported cases (black), termed IPs (although we note that some infected farms may have been culled before they were reported and some farms reporting the disease may not have been infected), and the number of culled farms (grey), including DCs, CPs, SOS, local and 3 km culls. In addition, figure 2*a* also marks the onset of different national control measures. This graph therefore provides a time frame against which the changing measures of model accuracy can be gauged. Figure 2*b* shows a typical temporal result from simulations, which is in qualitative agreement with the patterns of figure 2*a*.

Figure 2*c*–*h* shows the accuracy (solid lines) and repeatability (dashed lines) for the whole of the UK. Figure 2*c*,*e*,*g* shows the results starting from 23 February and simulated until various end times; as such, these illustrate the precision of models initiated early in the epidemic and show how the accuracy changes as longer time periods are considered. By contrast, figure 2*d*,*f*,*h* illustrates intervals of two weeks with different starting points, and hence shows how the predictive accuracy varies over the course of the epidemic. Figure 2*c*–*h* shows the accuracy of considering all farms, considering only farms reporting infection and considering only farms that are culled, respectively. Simulations started on 23 February use the precise conditions at that time as estimated from the future notification of cases. The simulations started at later times only use the reported cases and culls to that date to inform the initial conditions; this is necessary as the current infectious status of farms is the main short-term predictor of reported cases. These results may be heavily influenced by control policies in individual regions. Equivalent results for the five regions are given in the graphs in the electronic supplementary material. The same behaviour is found for each region—the results are qualitatively the same but quantitatively different (see the electronic supplementary material).

Looking at the total accuracy (figure 2*c*,*d*), we observe that the peaks in the cases in late March and early April are associated with a significant drop in accuracy (although the accuracy remains above 90%); however, this reduction is mirrored by a similar change in the repeatability, suggesting that it is primarily associated with between-epidemic variability during this period. We note that in both figures, the mean accuracy values are close to, but generally just outside, the 95% CIs of the repeatability values; the remaining figures examine the source of this inaccuracy. The model predicts the identity of reported cases (accuracy_reported_(23 February, end)) with an accuracy of just 12%, i.e. starting on 23 February the model correctly identifies one in eight farms that will be infected over the entire course of the epidemic (figure 2*e*). Examining the short-term predictive accuracy (figure 2*f*, accuracy_reported_(*t*_0_, *t*_0_+14)) reveals that less than 10% of the cases are correctly identified over a two-week period. However, these low values are consistent with the levels of between-epidemic variability predicted by the model (13.5% compared with 12% in figure 2*e* and 4.5–14% compared with 4–10% in figure 2*f*). Hence, according to our model, the highly stochastic nature of disease transmission means that the short-term future can only be predicted with very limited accuracy; greater accuracy occurs when considering the entire epidemic as determining risk factors play a far larger role.

Table 3 gives odds ratios for the entire epidemic, from 23 February until the disease is eradicated. The second column lists odds ratios for model simulations against the 2001 data, while the third column provides the associated model–model comparison; given ranges encompass 95% of all simulations. We see that, for the whole country and across all the five regions individually, odds ratios are always found to be greater than 1. As expected from equation (3.4), the odds ratio is generally the smallest in those regions that suffered the greatest proportion of reported cases, hence the highest values occur for Scotland and the rest of England (excluding Cumbria and Devon), while Cumbria yields the lowest value of odds ratio. Comparing the model–data and model–model odds ratios for the various regions, we naturally find that the model–model values are higher; however, the values for the main foci of infection are in close agreement, strengthening our belief in the accuracy and predictive benefit of the model in these regions.

Table 4 summarizes the short-term predictive ability of the model, by comparing the two-week intervals with the start dates (and hence initial conditions) spaced over the entire epidemic. The table provides mean odds ratios averaged across all the start dates. Clearly, the mean odds ratio will vary with the start date and this variation is captured by the minimum and maximum values. The minima generally occur when there are very few cases, and mean odds ratios less than one are found to coincide with extinction or introduction of infection into a region. Owing to the short time scales involved, the odds ratios are frequently higher than those given in table 3, indicating a greater predictive ability.

Turning our attention to the prediction of culled farms (figure 2*g*,*h*), we find the somewhat surprising result that although the levels of accuracy (solid lines) are higher (approx. 20–25%), these are not comparable with the levels of repeatability (dashed lines), which can exceed 60% in the short term. Our model–model comparisons therefore predict that culls should be readily predictable in the short term, which is consistent with the notion of a fixed control policy. However, the situation on the ground was far more complex, with judgements being made on a case-by-case basis. We believe it is in part this ‘unpredictable’ human element that causes the relative lack of accuracy. Developing models that can simulate the human response to perceived epidemic risk is vitally important for long-term predictions of both the livestock and the human infections.

## 5. Multiple simulations

The comparisons so far have been between individual replicates and the 2001 epidemic—hence our results are strongly influenced by the stochastic nature of the simulations. An alternative approach is to consider the results of multiple simulations and use the proportion of simulations in which a farm is infected (or culled) as a measure of its risk. These results are shown in figure 3 for simulations of the entire epidemic beginning on 23 February. Figure 3*a* shows the distribution of the proportion of simulations in which a given farm reports infection; this is partitioned into those farms reporting infection in 2001 (grey) and those not (white). Clearly, the two distributions are very different, with the distribution for farms reporting in 2001 showing a distinct secondary peak. This is an additional evidence that the simulations can partially discriminate between those farms that are likely to become infected and those that are not. Figure 3*b* shows comparable results for farms culled in the simulations and in 2001; here the distributions are more similar and this re-enforces our belief that human influence in the culling policy makes it far more difficult to simulate.

Finally, we can use the results of multiple simulations to ascertain if we can improve the predictive accuracy of our model. The basic concept is to determine a threshold proportion of simulations *P*_c_, such that farms reporting infection in more than *P*_c_ simulations are considered as likely to report infection in 2001, whereas those that report infection in less than *P*_c_ simulations are likely to remain susceptible—noting that this may change the number of cases predicted. Figure 3*c* shows the number of correctly identified cases together with the number of false-positive and false-negative errors as the threshold proportion is varied. Very low thresholds mean that we correctly identify the overwhelming majority of cases in 2001, but this is at a cost of many false positives. At the other extreme, when the threshold is very high, although there are few false positives, many farms reporting infection in 2001 are not identified. Three key threshold values can be identified. Values of *P*_c_ approximately 12% lead to half of all cases being identified, although a further 4000 false positives are predicted. Taking *P*_c_=14% minimizes the total number of errors (both false positives and false negatives) relative to the number of correctly identified farms reporting infection (figure 3*d*). Finally, setting *P*_c_=17.5% leads to predicted epidemics of approximately 2026 cases (agreeing with the size of the 2001 epidemic); for this threshold, a quarter of all cases are correctly identified showing the increased accuracy that can be gained by averaging over multiple model epidemics.

## 6. Discussion

Determining what constitutes a good model is often a value judgement. In general, we require a model that can predict the trends and patterns which are considered important, while other features are often deemed spurious. For example, we may stipulate that a model must capture the pattern of cases in the dominant epidemic regions but we may be less concerned about correctly identifying individual isolated cases. Initial parametrization of the model (Keeling *et al*. 2001, 2003; Tildesley *et al*. 2006) reflects this emphasis: attempting to match the number (and approximate timing) of cases and culls in five regions, while ignoring precisely which farms were involved. The parametrization method used in this paper also accounts for the cumulative number of livestock lost; this extra degree of fit is made possible by the inclusion of power laws in the rate of infection accounting for the nonlinear effects of livestock number. We find that while assuming a linear relationship between susceptibility and livestock numbers provides a good fit to the number of farms affected in the 2001 epidemic, it slightly overestimates the number of animals affected as infection is biased towards larger farms. Introducing powers into the model reduces the effect of animal numbers and therefore the average size of a farm infected decreases. We therefore find that this re-parametrization provides a close fit to the 2001 epidemic in terms of both the number of farms and the number of animals affected. In general, the inclusion of power laws also improves the accuracy with which the status of individual farms is predicted, although the general patterns and qualitative conclusions hold.

In contrast to this aggregate approach to parametrization, the measures of accuracy discussed in this paper are individual based, reflecting the ability to identify reported and culled farms correctly. As such, this acts as an independent verification of the model and its parametrization. The simplest measure of accuracy—proportion of farms correctly predicted to be reported, culled or unaffected (susceptible)—consistently produced extremely high accuracy values owing to the overwhelming number of unaffected farms. We therefore focus on the accuracy of predicting reported cases and culls separately, which are the key features of interest. It is important to note that these measures in themselves are not sufficient, as a model that overestimates cases and culls would have a high accuracy (high sensitivity but low specificity); however, given that our model has been shown to match the observed temporal pattern (in terms of predicting the number of cases and culls), this is not an issue.

Our analysis reveals that although the accuracy of predicting cases appears low (at approx. 10–15%), this should be considered relative to the model repeatability that provides a similar statistic for the agreement between two model replicates and therefore accounts for stochastic variability between epidemics. In general, we find that accuracy and repeatability are in close agreement and we see that the model does a remarkably good job of capturing the observed temporal pattern of the epidemic and the spatial pattern of cases at the farm level despite the fact that parametrization is based on aggregate information at a larger scale. If our sole aim is to identify the farms that are likely to become infected, then the results of multiple simulations can be further used beneficially to improve model prediction at the risk of generating more false positives—we can improve sensitivity but only at the cost of reduced specificity.

We now turn to the issue of what could be done to improve the performance of the epidemiological model. This can be partitioned into two separate elements: improvements to the model and the data quality and availability. Several improvements to the model can be readily envisaged. We currently use power-law scaling to capture the nonlinear relationship between the number of livestock and the risk of infection or transmission; other nonlinear relationships could be considered, although each would entail further re-parametrizations. The single transmission kernel could be replaced by two kernels, one for each species, or even four different kernels accounting for the different species–species interactions; in addition, different kernels could be used to account for differences between infection and DC detection. Extra detail could also be included for the within-farm dynamics. However, Savill *et al*. (2007) has investigated this issue and concluded (due to lack in data quality from the 2001 epidemic) that there is no evidence of changing infectiousness over infectious period of a farm. The inclusion of such additional heterogeneities as described above is likely to reduce the between-epidemic variability as it will generally increase our specification of identifying at-risk farms.

It is interesting to contrast the predictive accuracy of reported cases with that of culling. The model appears slightly biased towards correctly predicting IPs but not DCs. While the chance of correctly predicting an individual cull is greater, there is a higher discrepancy between the accuracy (model versus data) and the repeatability (model versus model). These two somewhat contradictory observations can be explained as follows. The higher levels of accuracy for culls compared with cases are primarily due to the higher number of culls compared with reported cases. However, we believe that the relatively lower value of accuracy compared with repeatability is due to the complexities underlying the true culling process. In reality, the decision to cull a farm is based upon a large range of value judgements that determine both national policy and local implementation in response to the current epidemic situation. By contrast, within our model, the implementation of specific strategies (such as the decision to CP cull or to introduce rapid culling of IPs and DCs) occurs at fixed times—this is expected to lead to less variation between individual model replicates. This also highlights a fundamental issue with the mathematical modelling of disease and control; while the dynamics of disease spread may be governed by a few relatively simple rules, the level and types of control applied are based on complex human value judgements which may be difficult to simulate.

In conclusion, we have shown that although the model is parametrized by matching to regional-scale dynamics, there is still relatively good agreement between the model replicates and the 2001 epidemic data at the individual farm scale. In particular, the epidemiological transmission of infection is predicted with an accuracy comparable to that between two model replicates. Much of the disagreement can be attributed to the stochastic chance nature of transmission and the fact that any two independent epidemics are therefore inherently different. We therefore conclude that these results support the use of this type of model as a predictive tool for retrospective analysis of the 2001 epidemic and for ascertaining the success of alternative strategies against future outbreaks—although refinements based on the inclusion of more biologically realistic processes may improve the model further.

## Figures and Tables

**Figure 1:**
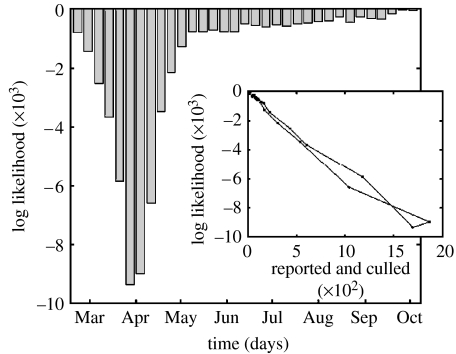
Graph showing the log likelihood of correctly predicting the status of all farms in a one-week interval for varying start dates. Likelihoods are calculated independently for each farm, from the results of multiple stochastic simulations. Farms are defined as being in the correct class if they are infected or culled (or simply remain susceptible) in both the model and the 2001 data in a given one-week prediction interval. The inset shows the log likelihood against the total number of reported and culled farms for each starting point of the simulations—we note that the log likelihood increases linearly with the number of reported and culled farms.

**Figure 2:**
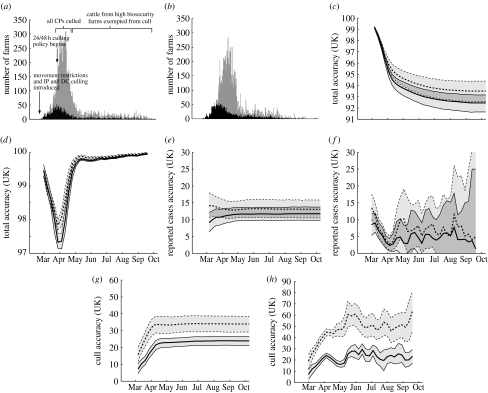
Model and data comparison for the entire country. (*a*) The daily number of farms that report infection (black) and farms that were culled (grey), together with the timings of national control measures for the 2001 epidemic. (*b*) Similar results from a single replicate model simulation, starting with the conditions on 23 February 2001. (*c*–*h*) Accuracy (solid lines) and associated repeatability (dashed lines) results (together with 95% CIs) for various time intervals and various farm types. If *t* is the time on the *x*-axis, the accuracy results are (*c*) accuracy_all_(23 February, *t*), (*d*) accuracy_all_(*t*, *t*+14), (*e*) accuracy_reported_(23 February, *t*), (*f*) accuracy_reported_(*t, t*+14), (*g*) accuracy_culls_(23 February, *t*), (*h*) accuracy_culls_(*t*, *t*+14). At least 2500 simulations were used to determine each data point. Regional plots, for Cumbria, Devon, the rest of England, Wales and Scotland, are shown in the electronic supplementary material.

**Figure 3:**
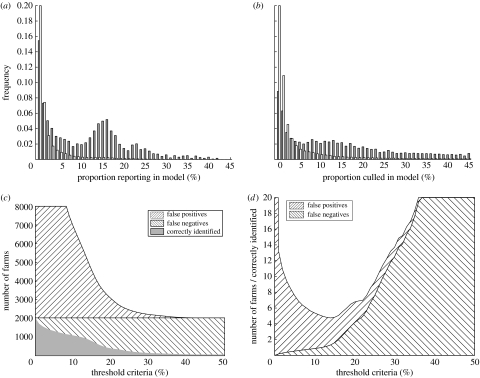
Results of multiple simulations of the entire epidemic for the whole of Great Britain. (*a*) The distribution of farms reporting infection in proportion *p* of simulations. This distribution is partitioned into those farms reporting infection in 2001 (grey) and those not (white). (*b*) Comparable distributions for culled farms again partitioned into those farms culled in 2001 (grey) and those not (white). In graphs (*c*,*d*), we define a threshold proportion *P*_c_, such that only those farms reporting infection in more than *P*_c_ simulations are identified as likely to report infection in the 2001 epidemic. (*c*) The number of correctly identified reports (grey) and the number of false positives and false negatives (hatched lines). (*d*) The number of false positives and false negatives (hatched lines) relative to the number of correctly identified reports. (Results are from 250 replicate simulations.)

**Table 1:** Values for the seven epidemiological factors for Cumbria, Devon, the rest of England, Wales and Scotland.

parameter	Cumbria	Devon	rest of England	Wales	Scotland
*S*_cow_	5.7	4.9	2.3	0.7	10.2
*T*_sheep_(×10^−4^)	8.3	11.0	23.2	36.3	28.2
*T*_cow_(×10^−4^)	8.2	5.8	8.2	30.1	23.2
*p*_s_	0.20	0.40	0.30	0.43	0.33
*p*_c_	0.41	0.37	0.42	0.31	0.23
*q*_s_	0.49	0.42	0.37	0.22	0.40
*q*_c_	0.42	0.37	0.44	0.25	0.20

**Table 2:** The mean value (and 2.5 and 97.5 percentiles) for the matrix of nine variables that record the number of farms in a particular state in the 2001 data and in the simulated model outbreaks. (Due to the large number of simulations involved the CIs for the mean are very small; therefore percentiles are quotes such that 95% of the simulations lie within the given range. The diagonal elements give the total number of farms whose status is correctly predicted by the model.)

data	model		

	reported	culled	susceptible
reported	230 (193–269)	733 (666–795)	995 (920–1081)
culled	519 (436–604)	1962 (1738–2167)	5703 (5438–5986)
susceptible	1323 (977–1699)	4977 (3785–6318)	171 982 (170 293–173 513)

**Table 3:** Odds ratios for reported farms over the entire epidemic. (Again, owing to the small CIs about the mean, 2.5 and 97.5 percentiles are quoted such that 95% of the simulations lie within the given range. Results are given for the whole of Great Britain and for the five regions.)

region	odds ratio (model–data)	odds ratio (model–model)
whole GB	13.41 (10.77–16.66)	15.61 (12.05–20.16)
Cumbria	1.91 (1.51–2.42)	1.93 (1.51–2.53)
Devon	4.02 (1.41–8.84)	4.86 (1.62–15.85)
rest of England	9.01 (5.33–13.61)	17.64 (11.37–28.32)
Wales	5.66 (1.78–20.22)	11.39 (2.83–62.78)
Scotland	26.06 (15.91–40.79)	27.42 (16.20–45.43)

**Table 4:** Mean odds ratios for reported farms over a two-week interval averaged over different start dates. (The maximum and minimum odds ratios correspond to average values at specific start dates and therefore capture the variation across the course of the epidemic and not between epidemic simulations. Again results are given for the whole of Great Britain and for the five regions.)

region	model–data odds ratio		model–model odds ratio	
	mean	(min–max)	mean	(min–max)
whole GB	208.39	(15.25–998.67)	322.68	(18.89–1437.01)
Cumbria	13.79	(2.01–83.15)	18.53	(2.49–119.27)
Devon	13.41	(0.46–129.72)	43.95	(4.72–381.64)
rest of England	188.26	(5.59–1458.62)	281.93	(30.35–1143.65)
Wales	93.54	(0.48–968.54)	171.79	(13.92–709.96)
Scotland	83.48	(2.54–735.64)	848.73	(23.10–1101.47)

## Supplementary Material

Additional material Supplementary material showing model accuracy and repeatability for the five regions: Cumbria, Devon, the rest of England, Wales and Scotland

## References

[bib2] Anderson I (2002). Foot and mouth disease 2001: lessons to be learned enquiry.

[bib1] Anderson R.M, May R.M (1983). Vaccination against rubella and measles—quantitative investigations of different policies. J. Hyg.

[bib4] Ferguson N.M, Donnelly C.A, Anderson R.M (2001a). The foot-and-mouth epidemic in Great Britain: pattern of spread and impact of interventions. Science.

[bib5] Ferguson N.M, Donnelly C.A, Anderson R.M (2001b). Transmission intensity and impact of control policies on the foot and mouth epidemic in Great Britain. Nature.

[bib6] Ferguson N.M, Keeling M.J, Edmunds W.J, Gani R, Grenfell B.T, Anderson R.M, Leach S (2003). Planning for smallpox outbreaks. Nature.

[bib3] Ferguson N.M, Cummings D.A.T, Cauchemez S, Fraser C, Riley S, Meeyai A, Iamsirithaworn S, Burke D.S (2005). Strategies for containing an emerging influenza pandemic in Southeast Asia. Nature.

[bib7] Ferris N.P, King D.P, Reid S.M, Shaw A.E, Hutchings G.H (2006). Comparisons of original laboratory results and retrospective analysis by real-time reverse transcriptase-PCR of virological samples collected from confirmed cases of foot-and-mouth disease in the UK in 2001. Vet. Rec.

[bib9] Hall I.M, Egan J.R, Barrass I, Gani R, Leach S (2007). Comparison of smallpox outbreak control strategies using a spatial metapopulation model. Epidemiol. Infect.

[bib10] Honhold N, Taylor N.M, Wingfield A, Einshoj P, Middlemass C, Eppink L, Wroth P, Mansley L.M (2004). Evaluation of the application of veterinary judgement in the pre-emptive cull of contiguous premises during the epidemic of foot-and-mouth disease in Cumbria in 2001. Vet. Rec.

[bib11] Kao R.R (2003). The impact of local heterogeneity on alternative control strategies for foot-and-mouth disease. Proc. R. Soc. B.

[bib12] Keeling M.J (2001). Dynamics of the 2001 UK foot and mouth epidemic: stochastic dispersal in a heterogeneous landscape. Science.

[bib13] Keeling M.J, Woolhouse M.E.J, May R.M, Davies G, Grenfell B.T (2003). Modelling vaccination strategies against foot-and-mouth disease. Nature.

[bib14] Kitching R.P, Hutber A.M, Thrusfield M.V (2005). A review of foot-and-mouth disease with special consideration for the clinical and epidemiological factors relevant to predictive modelling of the disease. Vet. J.

[bib15] Longini I.M, Halloran M.E, Nizam A, Yang Y (2004). Containing pandemic influenza with antiviral agents. Am. J. Epidemiol.

[bib16] Morris R.S, Wilesmith J.W, Stern M.W, Sanson R.L, Stevenson M.A (2001). Predictive spatial modelling of alternative control strategies for the foot-and-mouth disease epidemic in Great Britain, 2001. Vet. Rec.

[bib17] Savill N.J, Shaw D.J, Deardon R, Tildesley M.J, Keeling M.J, Woolhouse M.E.J, Brooks S.P, Grenfell B.T (2007). Effects of data quality on farm infectiousness trends in the UK 2001 foot and mouth disease epidemic. J. R. Soc. Interface.

[bib18] Thrusfield M, Mansley L.M, Dunlop P.J, Taylor J, Pawson A, Stringer L (2005). The foot-and-mouth disease epidemic in Dumfries and Galloway, 2001. 1: characteristics and control. Vet. Rec.

[bib19] Tildesley M.J, Savill N.J, Shaw D.J, Deardon R, Brooks S.P, Woolhouse M.E.J, Grenfell B.T, Keeling M.J (2006). Optimal reactive vaccination strategies for an outbreak of foot-and-mouth disease in Great Britain. Nature.

