# Supplementary material for: Accuracy of models for the 2001 foot-and-mouth epidemic
Source: Proc Biol Sci. 2008 Mar 25;275(1641):1459–68. doi: 10.1098/rspb.2008.0006 (PMC2376304; doi:10.1098/rspb.2008.0006)

Supplementary Material

In the main text of this paper, we show model and data comparisons for the entire country. Here we show the same figures, but for the five regions considered in the paper: Cumbria, Devon, Wales, Scotland and the rest of England (excluding Devon and Cumbria).

For each figure, graph (a) shows the daily number of farms that report infection infection (black) and farms that were culled (grey), together with the timings of national control measures for the 2001 epidemic. Graph (b) shows similar results from a single replicate model simulation, starting with the conditions on 23 February 2001. Graphs (c-h) show accuracy (solid line) and associated repeatability (dashed line) results (together with 95% confidence intervals) for various time intervals and various farm type. If t is the time on the x-axis, the accuracy results are (c) $\text{Accuracy}_{\text{All}}(23\text{ Feb}, t)$, (d) $\text{Accuracy}_{\text{All}}(t, t+14)$,(e) $\text{Accuracy}_{\text{Reported}}(23\text{ Feb}, t)$, (f) $\text{Accuracy}_{\text{Reported}}(t, t+14)$, (g) $\text{Accuracy}_{\text{Culls}}(23\text{ Feb}, t)$, (h) $\text{Accuracy}_{\text{Culls}}(t, t+14)$. At least 2500 simulations were used to determine each data-point.

Tables 1 and 2 below show the mean accuracy and repeatability values respectively for each region over the entire epidemic, with 95% confidence intervals shown in brackets.

Region	Total	Cases	Culls
Whole Country	92.6(91.7-93.3)	12.0(9.7-13.9)	23.0(20.4-25.9)
Cumbria	45.0(40.9-49.5)	16.2(14.8-22.0)	36.1(32.4-38.7)
Devon	86.8(81.7-90.4)	5.6(1.2-11.0)	12.0(5.1-20.1)
Rest of England	95.2(94.4-96.0)	5.0(2.5-7.4)	10.1(6.2-15.6)
Wales	95.5(92.8-96.8)	2.2(0.0-5.6)	9.1(3.0-17.1)
Scotland	94.7(93.3-95.3)	15.0(9.5-20.3)	36.0(28.1-43.4)

Table 1: Mean accuracy over the entire epidemic for the whole country and the five regions: Cumbria, Devon, the Rest of England, Wales and Scotland. Total accuracy (for cases, culls and susceptible farms), reporting accuracy and culling accuracy are all given. 95% Confidence Intervals are shown in brackets.

Region	Total	Cases	Culls
Whole Country	93.7(92.7-94.5)	13.5(10.3-15.4)	33.8(29.1-38.9)
Cumbria	56.1(53.8-59.5)	16.1(14.3-22.4)	48.8(44.1-54.0)
Devon	88.4(81.8-93.8)	7.8(1.6-14.8)	18.7(7.9-31.8)
Rest of England	95.8(94.8-96.9)	8.6(5.2-13.8)	19.9(10.1-29.0)
Wales	96.2(92.9-98.3)	4.8(0.0-13.6)	15.6(4.4-29.5)
Scotland	95.4(93.8-96.5)	15.1(9.4-21.2)	45.6(31.9-58.4)

Table 2: Mean repeatability over the entire epidemic for the whole country and the five regions: Cumbria, Devon, the Rest of England, Wales and Scotland. Total repeatability (for cases, culls and susceptible farms), reporting repeatability and culling repeatability are all given. 95% Confidence Intervals are shown in brackets.

Supplementary Figure 1 Model and data comparison for Cumbria.

Supplementary Figure 2 Model and data comparison for Devon.

Supplementary Figure 3 Model and data comparison for Wales.

Supplementary Figure 4 Model and data comparison for Scotland.

Supplementary Figure 5 Model and data comparison for England, excluding Devon and Cumbria.

Supplementary Figure 1: Model and data comparison for Cumbria.

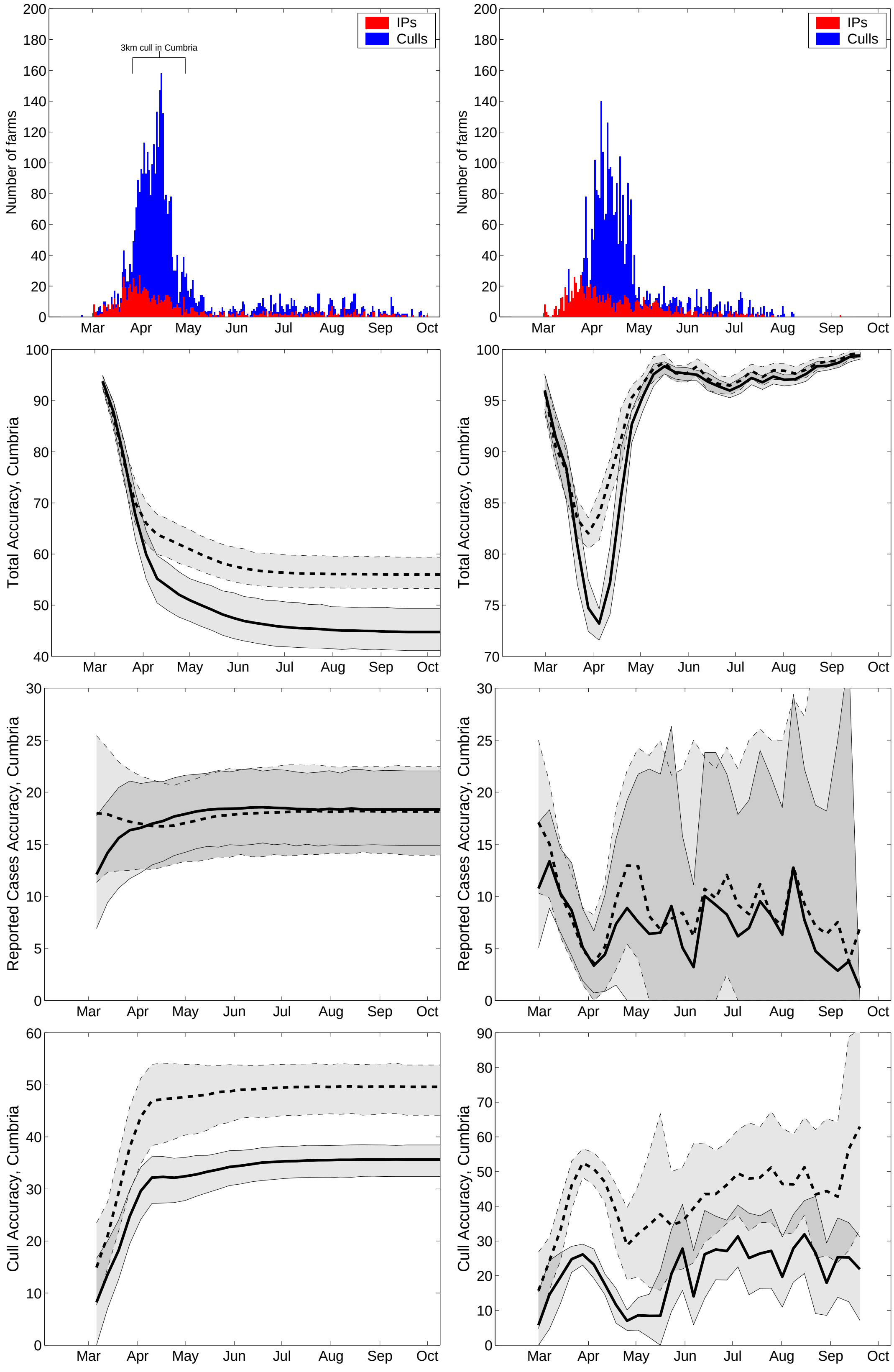

Supplementary Figure 2: Model and data comparison for Devon.

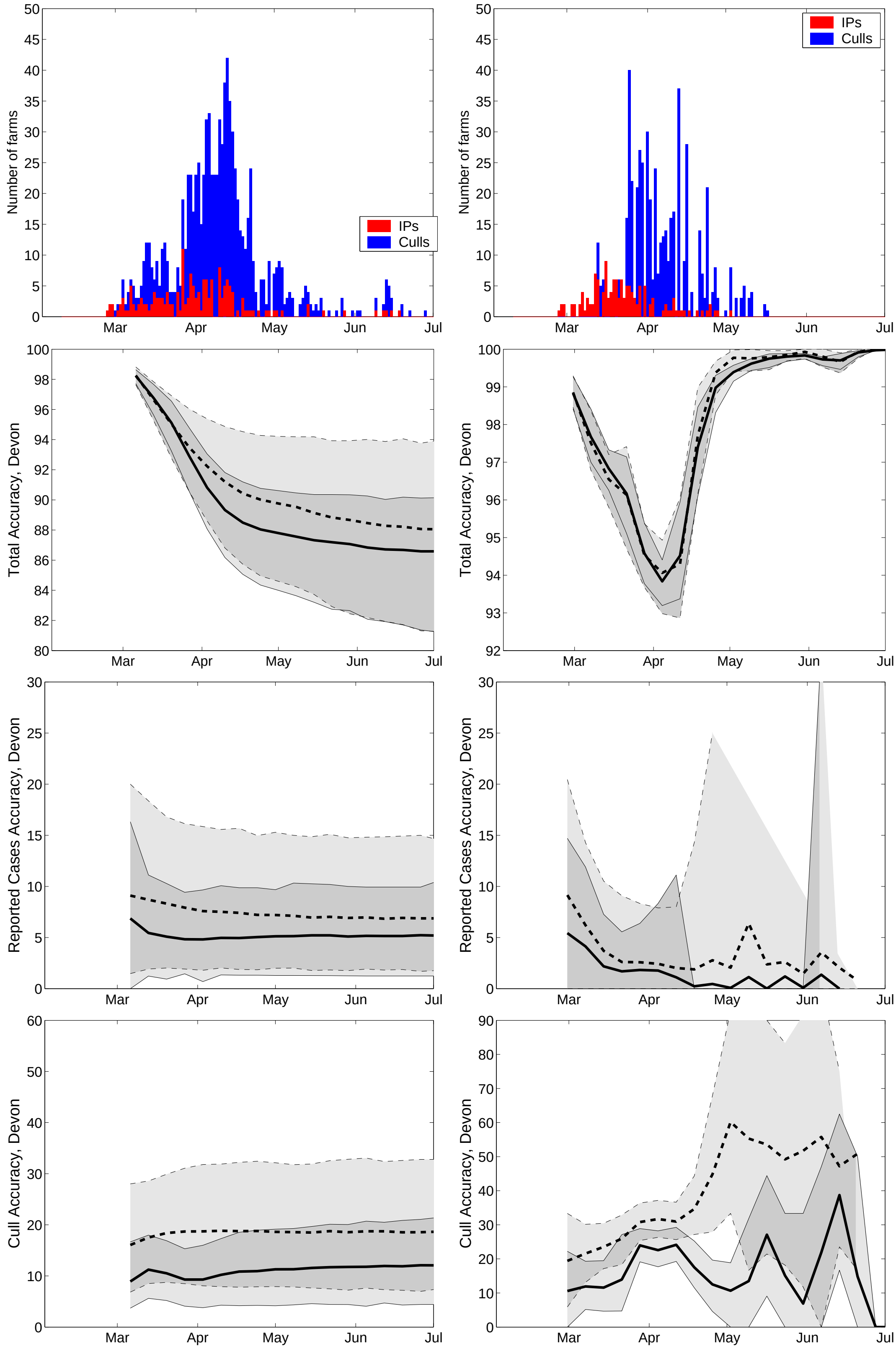

Supplementary Figure 3: Model and data comparison for Wales.

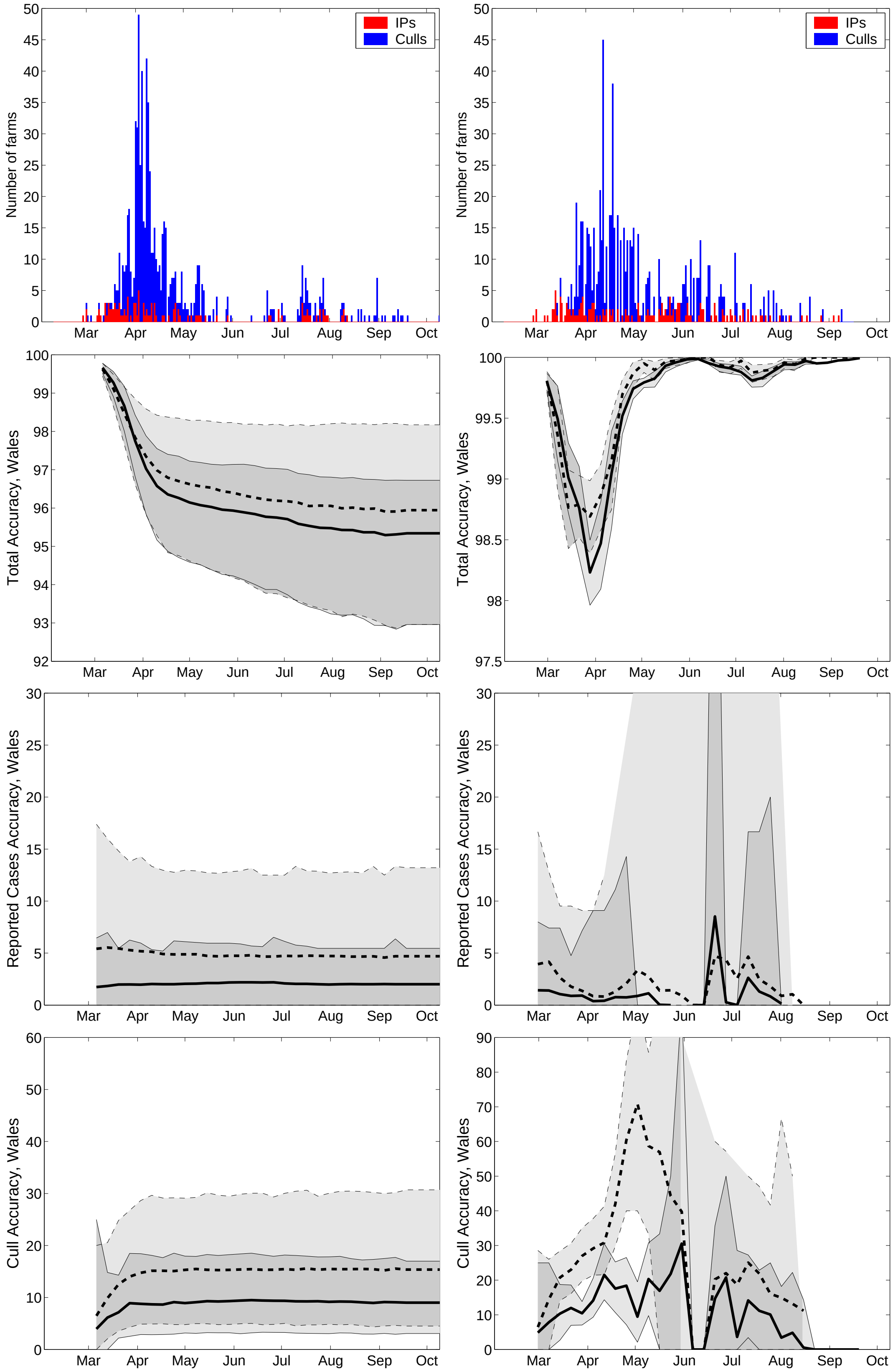

Supplementary Figure 4: Model and data comparison for Scotland.

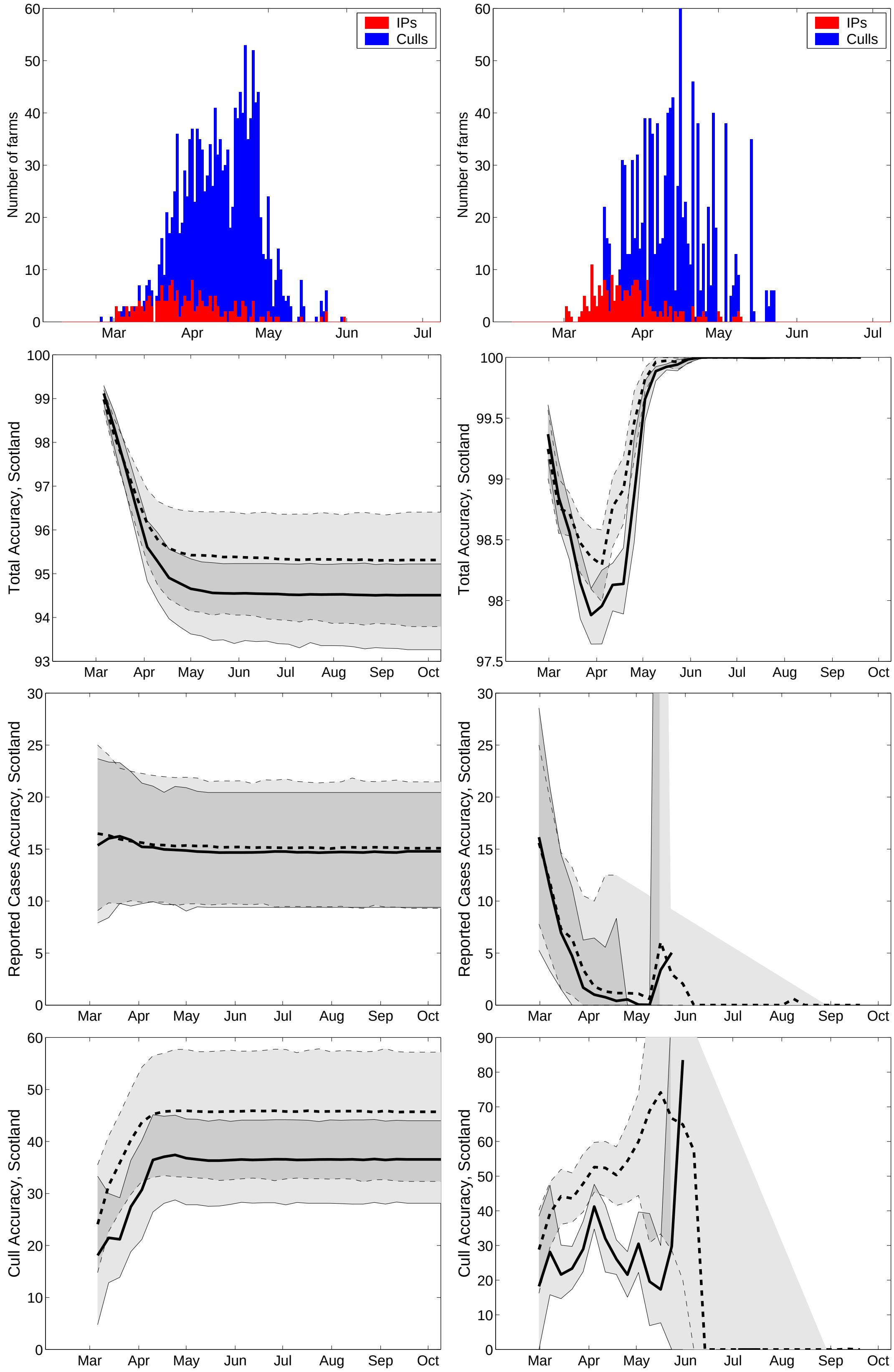

Supplementary Figure 5: Model and data comparison for England, excluding Devon and Cumbria.

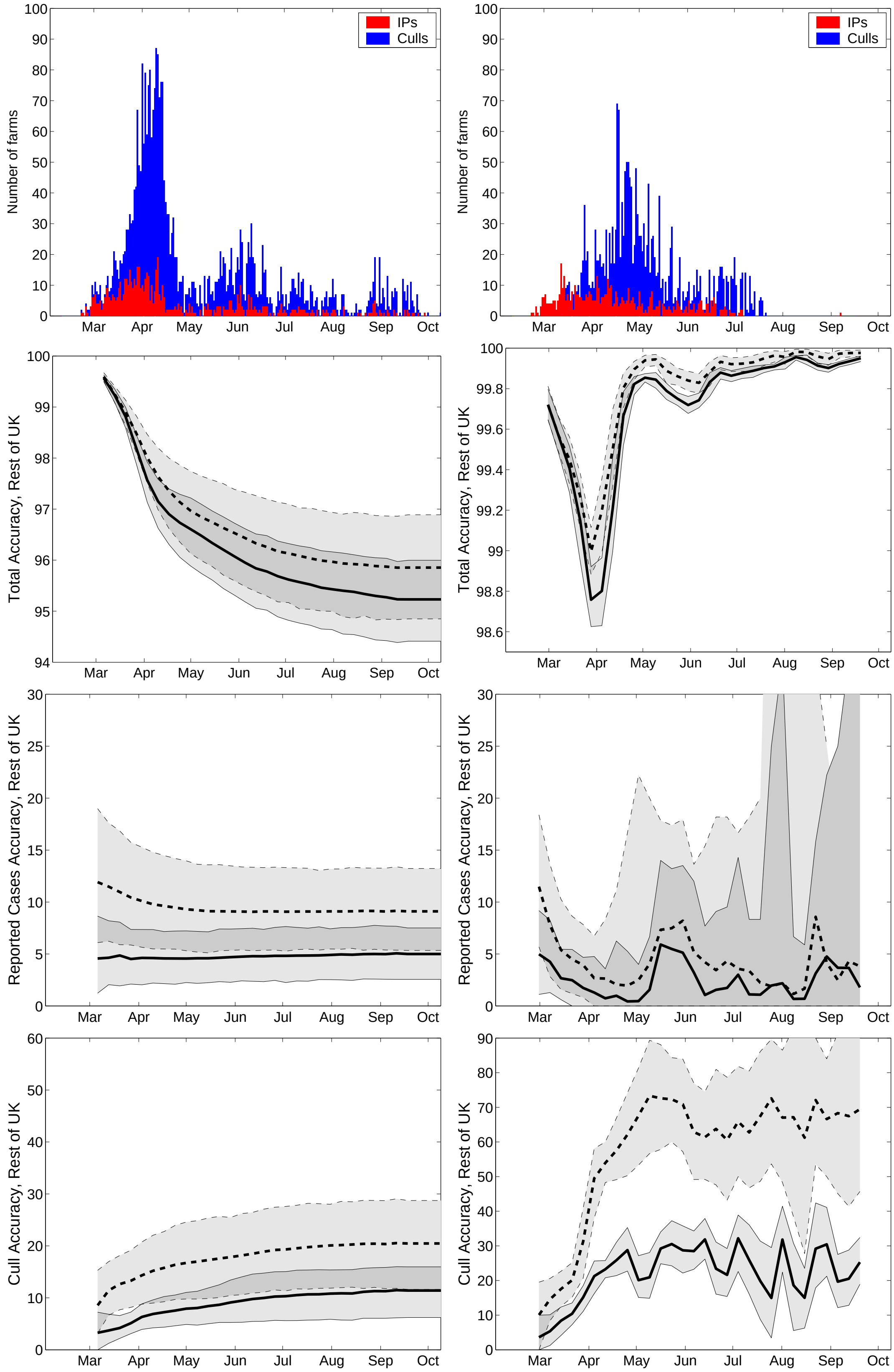

Supplement: Supplementary file 1 [file rspb20080006s09.pdf]
